# Computational solution of spike overlapping using data-based subtraction algorithms to resolve synchronous sympathetic nerve discharge

**DOI:** 10.3389/fncom.2013.00149

**Published:** 2013-10-31

**Authors:** Chun-Kuei Su, Chia-Hsun Chiang, Chia-Ming Lee, Yu-Pei Fan, Chiu-Ming Ho, Liang-Yu Shyu

**Affiliations:** ^1^Institute of Biomedical Sciences, Academia SinicaTaipei, Taiwan; ^2^Department of Anesthesiology, Taipei Veterans General Hospital, National Yang-Ming UniversityTaipei, Taiwan; ^3^Department of Molecular & Cell Biology, University of CaliforniaBerkeley, CA, USA; ^4^Department of Biomedical Engineering, Chung Yuan Christian UniversityChung Li, Taiwan

**Keywords:** spike sorting, spike overlapping, synchronous firing, spinal cord, autonomic nervous system, single-fiber recording

## Abstract

Sympathetic nerves conveying central commands to regulate visceral functions often display activities in synchronous bursts. To understand how individual fibers fire synchronously, we establish “oligofiber recording techniques” to record “several” nerve fiber activities simultaneously, using *in vitro* splanchnic sympathetic nerve–thoracic spinal cord preparations of neonatal rats as experimental models. While distinct spike potentials were easily recorded from collagenase-dissociated sympathetic fibers, a problem arising from synchronous nerve discharges is a higher incidence of complex waveforms resulted from spike overlapping. Because commercial softwares do not provide an explicit solution for spike overlapping, a series of custom-made LabVIEW programs incorporated with MATLAB scripts was therefore written for spike sorting. Spikes were represented as data points after waveform feature extraction and automatically grouped by *k*-means clustering followed by principal component analysis (PCA) to verify their waveform homogeneity. For dissimilar waveforms with exceeding Hotelling's T^2^ distances from the cluster centroids, a unique data-based subtraction algorithm (SA) was used to determine if they were the complex waveforms resulted from superimposing a spike pattern close to the cluster centroid with the other signals that could be observed in original recordings. In comparisons with commercial software, higher accuracy was achieved by analyses using our algorithms for the synthetic data that contained synchronous spiking and complex waveforms. Moreover, both T^2^-selected and SA-retrieved spikes were combined as unit activities. Quantitative analyses were performed to evaluate if unit activities truly originated from single fibers. We conclude that applications of our programs can help to resolve synchronous sympathetic nerve discharges (SND).

## Introduction

A challenge in elucidating the fundamental principles underlying the operation of a complex system such as the central nervous system is to achieve a measurement with signal resolution in both micro- and macroscales. The multielectrode recording techniques have been shown as a powerful tool to elucidate functional operations of cerebral cortical neurons (deCharms et al., 1999; Nicolelis et al., 2003; Tsytsarev et al., 2006; Galashan et al., 2011) and exemplify ensemble neuronal activities underlying somatic motosensory controls (Saleh et al., 2010; Vargas-Irwin et al., 2010). Applications of voltage-sensitive dyes in brain slice preparations also provide another mean to examine surface signals generating from the neural tissues and enable researchers for simultaneous examination of multiple signals at a resolution of single neuronal activities (Carlson and Coulter, 2008; Nakamura et al., 2008). However, the applicability of these techniques to resolve peripheral nerve activities at single-fiber levels is questionable.

The sympathetic nervous system is a vital neural network that controls many visceral functions through its direct innervation of different target organs. Effective visceral control largely depends on the central sympathetic commands being carried out to the periphery by the efferent fibers of sympathetic preganglionic neurons (SPNs) located in the thoracolumbar spinal cord of the vertebrates. The command signals are manifested by a basal sympathetic nerve discharge (SND), which often displays synchronous bursts (Iigaya et al., 2012; Steinback and Kevin Shoemaker, 2012; Fairfax et al., 2013). Conventionally, a direct measurement of SND is achieved using electrodes that make a direct contact with whole nerve bundles (Su et al., 1992; Jardine et al., 2002; Miki et al., 2002; Barrett et al., 2003; Marina et al., 2006; Orer et al., 2008; Madden and Morrison, 2009; Zahner et al., 2011); also see references in (Montano et al., 2009). The nerve signals are processed by electrical amplifiers and subsequently gauged in units of voltage. Although this conventional technique in recording the whole-bundle SND provides an easy assessment of the central sympathetic outflow, acquisition of detailed fiber activities could be crucial for understanding the elementary components of central sympathetic commands. Probably due to the technical obstacles, only limited studies have acquired unitary sympathetic fiber activities (Johnson and Gilbey, 1994; Macefield et al., 1994; Häbler et al., 1999; Lambert et al., 2006; Tan et al., 2009; Tanaka et al., 2009). Moreover, fewer studies have employed adequate algorithms to evaluate if the “unitary” activities are truly recorded from “one” single-fiber.

Our goal here is to establish an efficient way to record several sympathetic fiber activities simultaneously. We used a splanchnic sympathetic nerve-thoracic spinal cord preparation that can spontaneously generate rhythmic SND *in vitro* as an experimental model (Su, 1999). Collagenase was used for *in situ* dissociation of the splanchnic nerve bundles (Ho et al., 2013). The dissociated nerve fascicles were then brought into a suction electrode to record several fiber activities, the so-called “oligofiber recording.”

One main issue arose from the oligofiber recording techniques is that different sources of interference might add to the neural signals leading to a random variation of spike waveforms (Lewicki, 1998; Snider and Bonds, 1998). How to assign a nonstationary spike waveform to its originated fiber becomes an issue of spike sorting. We took advantage of LabVIEW softwares as proposed earlier by Stewart et al. (2004). In addition, we also took advantage of Matlab programs, which provide numerous mathematical functions for data probing. Programming based on a combination of both LabVIEW and Matlab renders us a greater flexibility for signal processing.

Computer analyses were based on the stochastic features of spike waveforms to group the neural signals that were likely originated from the same fiber. Two strategic methods were used sequentially for spike sorting. First, similar spike waveforms were automatically grouped by *k*-means clustering algorithm. Second, stochastically homogenous or ideal spike waveforms were extracted from each *k*-means cluster using principal component analysis (PCA) to represent data and using Hotelling's T^2^ distances as criteria to purge those data located distant from the cluster centroids. Although commercial softwares are available for spike sorting, they usually do not provide an explicit solution to decompose the complex waveforms resulting from spike overlapping. To further verify those complex waveforms with large T^2^ distances as true outliers, we used a subtraction algorithm (SA), which was conducted simply by subtracting an ideal spike waveform from the complex waveforms, followed by determining if the extracted, decomposed waveforms truly occur during the recording. This is a data-based approach and is not a mathematical approach as many other computational algorithms do. It is simply the best guess based on the signals obtained from original recordings to provide a possible but not the sole solution that the complex waveform may indeed occur because of spike overlapping. Applications of other computational algorithms also helped to evaluate if the seemingly “unit” activity was truly a single-fiber activity.

## Methods

### Animal

Experiments were performed using 30 neonatal Sprague-Dawley rats of age 3–5 postnatal days. All surgical and experimental procedures were approved by the Institutional Animal Care and Utilization Committee of Academia Sinica (Protocol#: RMiRaIBMSC2009064) in accordance with the Guide for the Care and Use of Laboratory Animals of the Agriculture Council of Taiwan.

### Splanchnic sympathetic nerve–thoracic spinal cord preparations *in vitro*

*En bloc* preparations retained the splanchnic sympathetic nerve–thoracic spinal cord (T1–T12) were prepared following surgical procedures as previously described (Su, 1999; Su et al., 2010). Briefly, neonatal rats were made unconscious by hypothermia (Danneman and Mandrell, 1997), followed by a prompt midcollicular decerebration. During dissection, the reduced preparation were immersed in ~4°C artificial cerebrospinal fluid (aCSF; in mM: 128 NaCl, 3 KCl, 1.5 CaCl_2_, 1.0 MgSO_4_, 24 NaHCO_3_, 0.5 NaH_2_PO_4_, 30 D-glucose, and 3 ascorbate; equilibrated with 95% O_2_–5% CO_2_). A stub of the splanchnic sympathetic nerves was freed from surrounding tissues and its distal end was severed adjacent to the celiac ganglion. The nerve-thoracic spinal cord preparation (T1–T12) was then immersed in a bath chamber containing 30-ml aCSF with temperature maintained at 24.5 ± 1°C. Dissociation of the nerve bundles was performed by incubating the splanchnic nerves for ~90 min in a glass micropipette containing 0.5% collagenase (Type IV collagenase, C5138, Sigma-Aldrich, buffered by Hanks' Balanced Salt Solution, 14185-052, Invitrogen Corporation). Dissociated nerve fascicles showing a nerve stub with split ends were easily obtained after the incubation (Ho et al., 2013).

### Neural recordings

Borosilicate glass micropipettes (AM-system, 5928, Carlsborg, Washington) were tapered using a horizontal puller (P-97, Sutter Instrument, Novato, California) to make long-shank recording electrodes with tips ~10 μm in diameter and back-filled with aCSF. Dissociated nerve fascicles were brought into the glass micropipette using a suction electrode (AM-system, 573000, Carlsborg, Washington) to record spontaneous spike potentials. Electrical signals were pre-amplified (DAM50; World Precision Instruments, Sarasota, Florida), amplified (NL106, Digitimer Ltd., Hertfordshire, England), bandpass filtered at 10–3000 Hz (NL126, Digitimer Ltd.), and stored on a pulse-code modulation tape recorder (Neuro-Corder DR-890; Cygnus Technology Inc., Delaware Water Gap, Pennsylvania). Analog signals were digitized in a real-time using a National Instrument-based data acquisition system (NI-PCI-6010, National Instrument, Austin, Texas) and processed using LabVIEW programs (version 8.2.1, National Instrument) incorporated with MATLAB scripts (version 7.9. The MathWorks, Inc., Natick, Massachusetts). To avoid aliasing and sampling jitter for precise waveform alignments at spike peaks, signals were first oversampled at 40 kHz and then downsampled to 10 kHz by interpolation algorithm to keep file size small. All signals were digitally corrected for amplification gains and expressed in units of μV for computational analyses.

### Spike detection and waveform feature extraction

**Figure 1:**
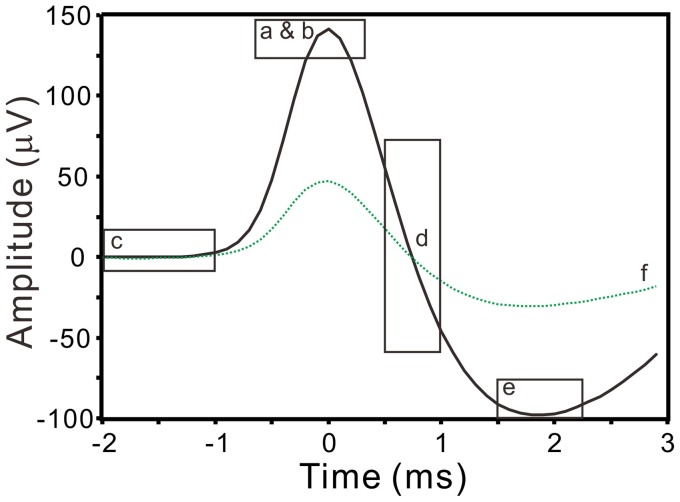
**Extraction of spike waveform features**. Squares enclose the waveform segments (solid line) that are used to evaluate spike peak amplitude (a), peak roundness (b), prespike amplitude (c), repolarization rate (d), afterhyperpolarization (e), and correlation coefficient (f) with a reference spike waveform (dashed line).

Off-line analyses of the recorded signals were performed using LabVIEW-based computer programs to analyze spiking events in a 30-min epoch of continuous recording in each experiment. Under our recording conditions, times for oligofiber spiking only took up a small fraction of the overall recorded signals (e.g., Spiking activity at 5 Hz with spike duration of 5 ms contributes to the signal with a ratio of 0.025 = 25 ms/1000 ms). Therefore, an automatic determination of thresholds for spike detection was based on an estimate of the standard deviation of background noise σ_*n*_ using the equation:

$$\sigma_{n}=\text{median}\left\{\frac{\left|x\right|}{0.6745}\right\}$$

where *x* is the band-pass filtered signal (10–3000 Hz); this yielded a robust estimation of σ_*n*_ that was relatively indifferent to the length of data segments being selected for analyses, because the median would reliably measure the instrumental noise of recordings (Quiroga et al., 2004). Spikes with peak amplitudes >5σ_*n*_ were automatically detected and the peak timing was taken as the timestamp of spike occurrence. Signals of 25-ms in duration extending from 12-ms prior to and 13-ms after the spike peak were aligned for spike waveform analyses. A reference spike waveform was constructed by averaging 20 spike waveforms of similar shape. To minimize interference, only the signal segments spanning from 10–15 ms (i.e., 2-ms prior to and 3-ms after the spike peaks) were used to extract spike waveform features. Waveform features were described by six parameters, including the peak amplitude, the peak roundness (i.e., the 2nd derivative of the spike peak), the root-mean-square of prespike amplitude (i.e., a magnitude measurement for a 1-ms signal segment starting from 2-ms prior to the spike peak), the afterhyperpolarization (i.e., afterspike minimum), the highest repolarization rate, and the coefficient correlated with the reference waveform (Figure 1). After feature extractions, a spike waveform was described by the acquired parametric values that could represent the waveform as a data point in a 6-D space (ℜ^6^).

### *k*-means clustering and principal component analysis

Automatic spike sorting was conducted first using a least square partitioning algorithm, the *k*-means clustering, which is mainly based on a calculation of *z*-scores (*z*) of the six waveform feature parameters obtained from the spikes occurred in 30-min epoch of continuous recording (Appendix A). For comparisons, in some experiments, *z*-values were obtained from a full-waveform calculation without a priori feature extraction. Therein, each amplitude value in the waveform segment extending from 1.2 ms prior to and 2 ms after the spike peak was taken as individual parametric value to calculate *z*-values for data representation in a space of ℜ^32^. Computation using cityblock distance and squared Euclidean distance for *k*-means clustering were compared in these experiments. To interpret the partitioning result and objectively evaluate the cluster separation, we used the silhouette technique (Kaufman and Rousseeuw, 1990) (Appendix B). After *k*-means clustering, apparent outliers with exceptionally large Hotelling's T^2^ distances in the cluster were removed automatically using the PCA to simplify data representation in multivariate space (Appendix C). In short, Hotelling's T^2^ distance is the multivariate counterpart of the Student's-*t*. The higher the T^2^ value represents more distance from the observation to the mean or the centroid. Homogeneity of principal component representations of the data was evaluated by their distance from the centroid of the cluster. Those data with Hotelling's T^2^ distances falling into a range exceeding 99.99% scope were considered as outliers (i.e., T^2^-unselected waveforms) and were subjected to empirical waveform evaluation (see below). In practice, *k*-means clustering and PCA were achieved simply by adding a MATLAB script node in the LabVIEW programs.

### Retrieval of T^2^-unselected waveforms using a data-based subtraction algorithm

In PCA, those data falling into a range exceeding 99.99% were considered as outliers, and collectively defined as T^2^-unselected spike waveforms. The outliers removed by PCA process were subjected to empirical waveform evaluation, based on a simple assumption that the distortion of the waveform was due to an addition of “noise” to the otherwise homogenous spike waveforms. First, a reference waveform from each cluster was obtained by averaging the spike waveforms in the cluster which had passed T^2^ selections. Second, residual waveforms in 25-ms duration were acquired after subtraction of the reference waveform from each T^2^-unselected waveform. Because feature extractions of the spike waveforms only evaluated a signal segment extending from 2-ms prior to and 3-ms after the spike peak, the maximum of the residual waveform occurring at 2–15 ms was detected and a 5-ms waveform segment spanning from 2-ms prior to and 3-ms after the maximum was extracted from the 25-ms residual waveform accordingly. This would extract an interference waveform that fell into the signal segment 10-ms prior to and 3 ms after original spike peaks. A LabVIEW program was written for automatically detecting the existence of the 5-ms residual waveforms in a 10-min epoch of the original recordings. Two criteria were used to recognize the occurrence of this “noise.” First, the maximum of the residual waveforms was <4σ_*n*_, i.e., <80% of the noise level of recording. Second, similar signals were found in the original recordings with correlation coefficient greater than 0.95 and with a maximal magnitude difference less than 30%. Upon fulfilling these criteria, the T^2^-unselected spike waveforms would be retrieved. For each *k*-means cluster, this semi-automatic evaluation process, the so-called “SA,” only required a subjective determination of the stringency for waveform selections to minimize false negative and to avoid false positive (see below). Both SA-selected and T^2^-selected data were combined and taken as unit activities.

### Evaluation of single-fiber activities

Neuronal firing behaviors may provide useful clues for spike sorting (Delescluse and Pouzat, 2006; Ventura, 2009). To implement an algorithm that can help to evaluate whether the unit activities acquired from the waveform-based algorithms are truly attributed to single fiber activities, the interspike intervals (ISIs) were taken into account. First, ISIs of values less than 3-ms were considered as a violation of refractory period (Horn and Friedman, 2003); the data incurring such violations were arbitrarily defined as “false positive.” Second, because ISI probability distribution in SPNs is nearly Gaussian (Su et al., 2007), we examined if this feature persisted in the data collected here. Third, because spike waveform features could be affected by previous spiking events, we examined if the waveforms features varied as a function of their preceding ISIs. To better reveal Gaussian curves in the data range of short ISIs, ISIs were transformed by natural log and categorized with a bin range from 3-ms (equivalent to *e*^−5.809^) to the upper 99.9% confidence limit of the ISI distribution. When appropriate, the plot of ISI probability distribution was constructed based on the bin width (*h*) of Scott's choice (Scott, 1979), as calculated by the equation:

$$h=3.49s\cdot n^{-1/3}$$

where *s* is the standard deviation of ISIs and *n* the number of ISIs. Probability distribution curves were fitted using the Gaussian equation:

$$y=\sum_{i=1}^{k}a_{i}\cdot e^{\left\{-1/2{\left[(x-b_{i})/c_{i}\right]}^{2}\right\}}$$

where *k* represents the number of modes 1–3, *a*_*i*_ the probability density (pd) at the mode, *b*_*i*_ the modes, and *c*_*i*_ is the half-maximal width. Evaluation of the best Gaussian fitting using different number of modes was based on the corrected Akaike information criterion (AIC_c_; Burnham and Anderson, 2002), calculated by the equation:

$${\text{AIC}}_{c}=n_{b}\cdot \ln(\text{SSR}/n_{b})+2K+\frac{2K(K+1)}{n_{b}-K-1}$$

where *n*_*b*_ the number of bins, SSR the sum of squared residuals obtained from error estimates in the particular Gaussian model, and *K* the number of parameters in the model. The best fitting was considered as the one yielded a minimal AIC_c_.

To examine whether changes of the waveform features were preceding ISI-dependent, waveform parametric values were plotted against their preceding ISIs in natural log-scales. Based on the pattern of data distribution, the curves were fitted by an exponential relaxation equation:

$$y=y_{0}+a\cdot e^{-(x\;-x_{0})/t}$$

where *a* the amplitude, *x*_0_ the center, *t* the relaxation time constant, and *y*_0_ the offset of the particular parameter and is equivalent to the parametric value at *x* → ∞ A negative value of *a* indicates that the magnitude of parametrical values is reduced when preceding ISIs become smaller. Alternatively, the curves were simply fitted by a linear equation:

y=mx+b

where *m* is the slope and *b* is the intercept. The best fitting, either by the exponential relaxation equation or by the linear equation, was again evaluated by AIC_c_.

### Data analysis

Spearman's correlation of coefficient (*r*) was acquired after plotting the observed data against the model predicted data in each curve fitting. Tests of goodness of fit by a model were performed using *r* to calculate Student's *t*-values (*t*) by:

$$t=r\cdot \sqrt{\left(n_{b}-K-1)/(1-r^{2}\right)}$$

where *n*_*b*_ is the number of bins, *K* the number of parameters in the model, and *n*_*b*_ − K − 1 the degree of freedom. A *P*-value <0.05 was considered significant. All values are expressed as mean ± SEM.

## Results

### Oligofiber activities recorded from collagenase-dissociated nerve fascicles

**Figure 2:**
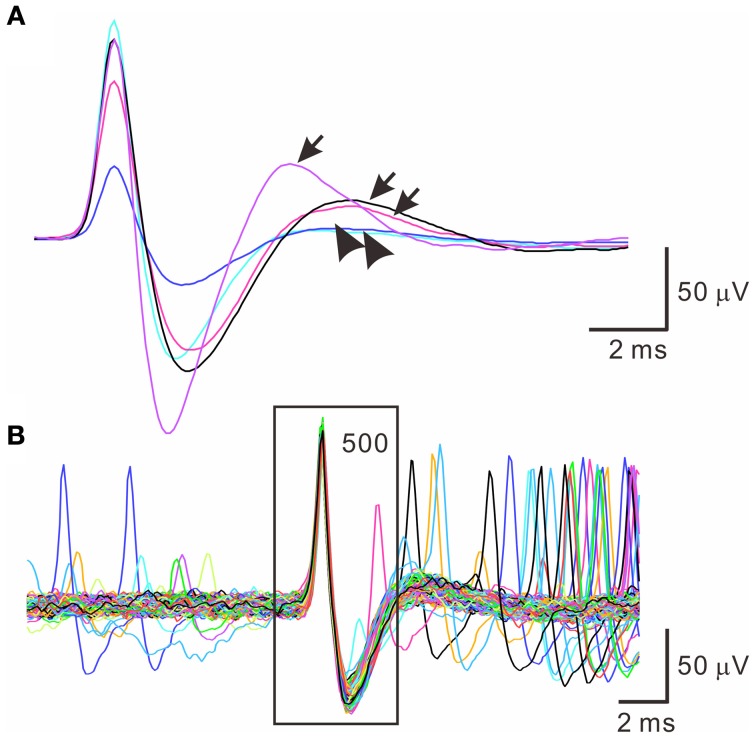
**Similar spike waveforms obtained from oligofiber recordings. (A)** Superimposed spikes showing similar waveform shapes. Traces are averaged spike waveforms obtained from different experiments. Spike waveforms are primarily biphasic. Arrows and arrow heads indicate the presence and absence of a rebound potential following the afterhyperpolarizations. **(B)** Superimposed traces of 500 spike waveforms showing the presence of small interference signals. Square encloses the waveform segment, 2-ms prior to and 3-ms after the spike peaks.

Spontaneous spiking signals were consistently obtained from electrical recordings of collagenase-dissociated nerve fascicles. A recording showing distinct spike waveforms may require several attempts in sampling different nerve fascicles. Recorded spike waveforms were primarily biphasic, showing an initial overshoot of the potential trajectory followed by a prompt fall and slowly-relaxed afterhyperpolarization. Figure 2 shows the common features of spike waveforms obtained from oligofiber recordings. Some spike waveforms had an afterdepolarization immediately followed the afterhyperpolarization. For computational comparisons, the spike waveforms were aligned horizontally at their peak and their waveform features were extracted accordingly. Because a collection of spike waveforms with longer durations was more likely to be contaminated by ambient electromagnetic interference and some background signals originated from spiking activities of other fibers (Figure 2B), the waveform feature extraction was limited to the early phase of spike waveforms as illustrated in Figure 1. Noticeably, not all the extracted features were equally effective to distinguish different spike waveforms. The usefulness of a waveform feature for spike sorting might vary between experiments. For instance, most of the waveforms were biphasic and had similar shapes. The similarity in waveform shapes did not permit the correlation coefficients of the spike waveforms with their reference waveform as an effective feature for spike sorting. Therefore, in some cases, the correlation coefficients were discarded as a waveform feature and were not used for the clustering analyses. Nonetheless, the correlation coefficients were found useful to distinguish signals of distinct waveforms, e.g., the waveforms of ambient interference from the waveforms of true neural signals.

**Figure 3:**
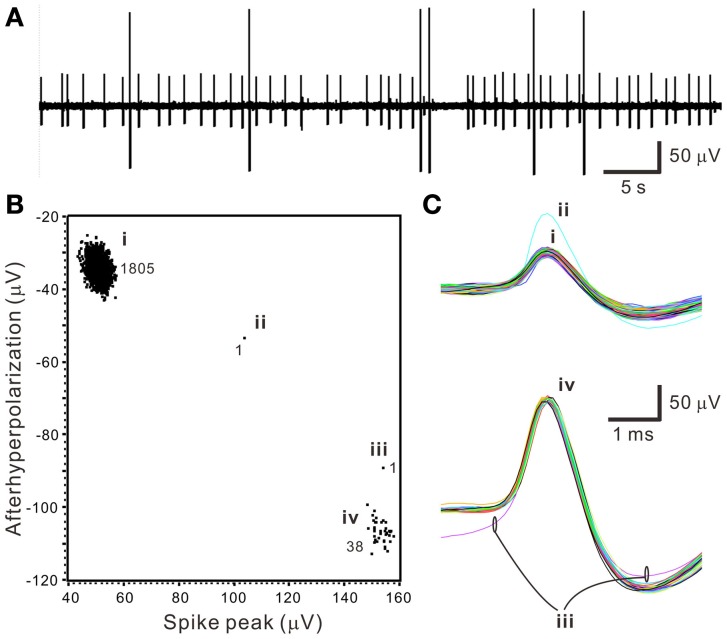
**Distinct oligofiber activities**. Data were collected from a 30-min epoch of continuous recording. **(A)** Original trace showing spikes of apparently different amplitudes. **(B)** Waveform feature plot showing segregated data distribution. Numerical values indicate the number of data points in groups (i–iv). **(C)** Spike waveforms of groups (i–iv). Data group (i) and (iv) forms apparent clusters.

The first step of spike sorting was constructing a 2- or 3-D plot that included the data points of interest by manually selecting any two or three waveform features clearly showing obvious cluster distribution patterns. Figure 3 shows an example that distinct oligofiber activities are recorded. Waveform parametric plot that showed at least one single-cluster distribution was successfully acquired in all experiments (*n* = 30). After selecting appropriate waveform parameters to construct the parametric plots, we identified 1.67 ± 0.18 clusters per experiment simply by visual inspection.

### *k*-means clustering of oligofiber activities

**Figure 4:**
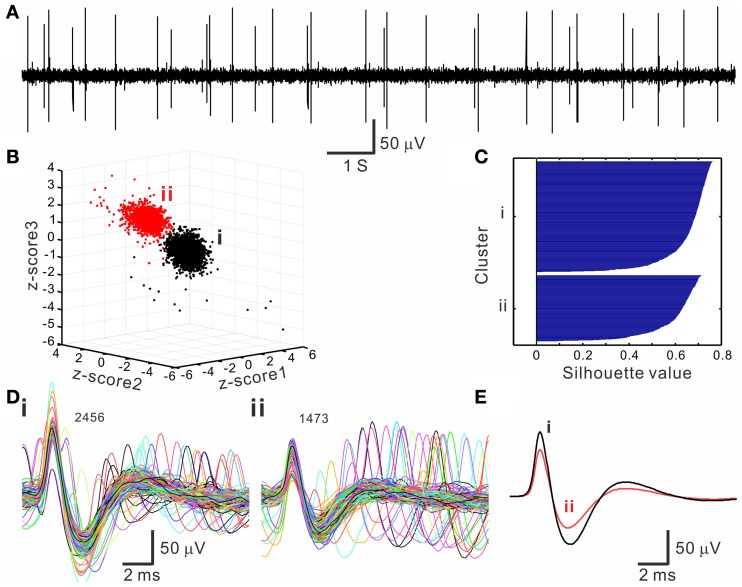
**Spike sorting by *k*-means clustering algorithms. (A)** An original trace showing oligofiber spiking. **(B)** 3-D *z*-score plot of waveform features showing two neighboring clusters (i, ii). *Z*-score 1–3 are normalized values of peak amplitude, peak roundness, and afterhyperpolarization. **(C)** Silhouette plot. Averaged silhouette value is 0.6571. **(D)** Spike waveforms in the *k*-means clusters (**i, ii**, as shown in **B**). Numbers of superimposed waveforms are as indicated. **(E)** Averaged spike waveforms of cluster (i, ii). Some waveforms as shown in (**D)** differed considerably from their averaged waveform.

**Figure 5:**
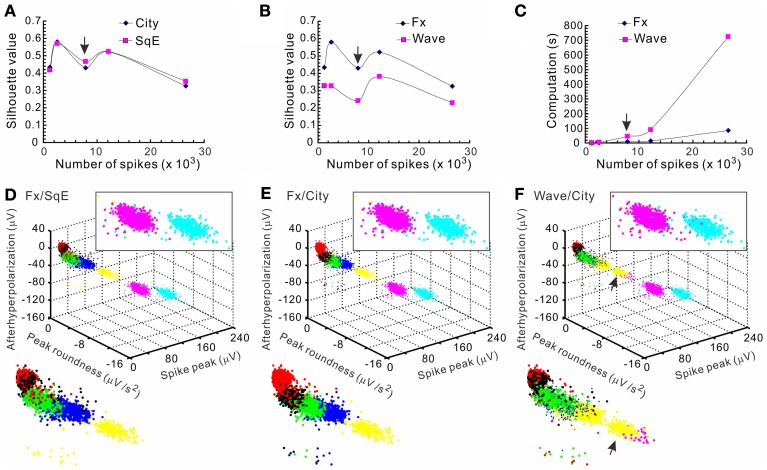
**Comparisons of the computational efficiency using different *k*-means clustering subalgorithms**. Clustering were based on different subalgorithms including cityblock distance (City), squared Euclidean distance (SqE), full spike waveform (Wave), and waveform feature extraction (Fx). Five data sets with each containing 1223–26585 spikes that formed 4–7 apparent clusters were compared. Clustering a data set containing 7871 spikes [arrows in **(A**–**C)**] is shown in **(D**–**F)**. The means of silhouette values were taken as a clustering goodness measure. **(A)** Comparisons of clustering based on City/Fx and SqE/Fx. Plots of the means of silhouette values against the number of spikes in the data set demonstrate that the goodness of clustering by City and SqE is largely comparable. **(B)** Comparisons of clustering based on Wave/City and Fx/City. Plots show that Fx-based clustering generates higher silhouette values. **(C)** Comparisons of computation times required for clustering based on Wave/City and Fx/City. Less computation time is needed for clustering with Fx. **(D–F)** 3-D plots of the data clusters obtained from different *k*-means subalgorithms. Colors code for the comparable clusters. A magnified view of data clusters is shown in the insets at the upper right and the lower left. Data clusters were obtained from the algorithms based on Fx/SqE **(D)**, Fx/City **(E)**, and Wave/City **(F)**. Upper right insets containing the two clusters of higher spike peaks show more perplexing cluster assignments in **(D)** and **(F)** than **(E)**. Also shown in the lower left insets, the arrows in **(F)** indicates a cluster containing data of misassignments.

The *k*-means clustering algorithm was used extensively for spike sorting, especially for data with distribution patterns that had no clear boundary. Figure 4 shows an example of *k*-means clustering that cluster boundaries are barely discernible. This algorithm was also helpful in automatic data clustering even when cluster boundaries were obvious. To make the *k*-means clustering more efficient, in experiments with spike numbers >10,000, the data forming apparent clusters in waveform parametric plots were manually removed to reduce computation times. Conventionally, *k*-means clustering is based on the squared Euclidean distance acquired from the parametrical values of a full spike waveform. Instead, we obtained the “cityblock” distance from the extracted waveform features. Figure 5 shows an example that compares the outcomes of spike sorting using different subalgorithms of *k*-means clustering. The operation using “cityblock distance” with waveform feature extraction was more computational efficient and yielded a clearer cluster separation than the one using squared Euclidean distance or the one with full spike waveforms.

The *k*-means clustering assigned each spike waveform to one of the clusters. Silhouette values were calculated for each spike and the resulted clusters were displayed on a silhouette plot (Figure 4C). A cluster in the plot showing equally high silhouette values is ideal because it represents the data in the cluster having similar waveform features. In 17 experiments that the cluster boundaries were obscure, the *k*-means clustering process increased the number of identifiable clusters by 2.48 ± 0.29. For the other 13 experiments, the *k*-means clustering did not increase the number of clusters because the cluster boundary was clear enough by visual inspection or because it failed in yielding clear clusters for those data points with obscure boundaries. On average, the number of clusters per experiment being identified after *k*-means clustering was 3.40 ± 0.38 (*n* = 30). The following analyses were based on these 102 data clusters.

### Selecting spike waveforms of homogeneous features

**Figure 6:**
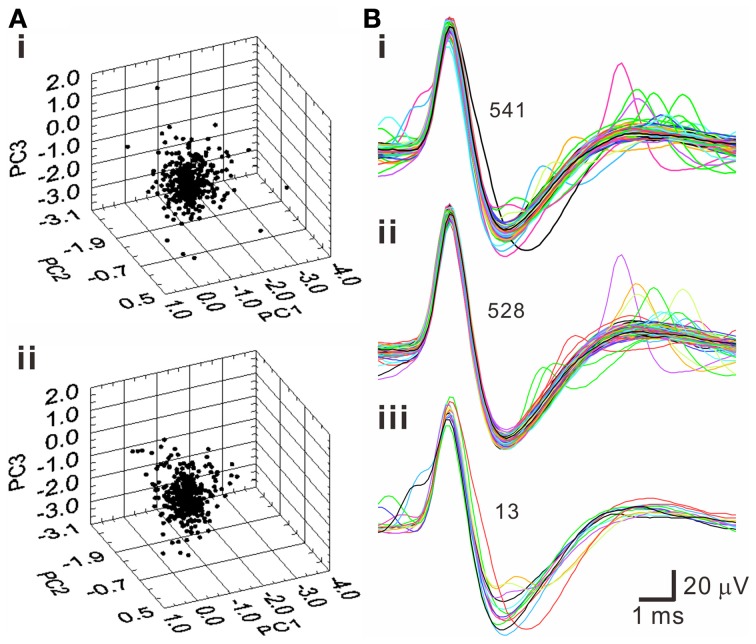
**Selection of homogenous spike waveforms by PCA algorithms using Hotelling's T^2^ distance as criterion**. **(A)** 3-D principal component (PC) plots. **(i)** Original plot. **(ii)** The plot after removing outliers. **(B)** Spike waveforms of the original data set **(i)**, the data set after removing outliers **(ii)**, and the outliers **(iii)**. Numbers of superimposed waveforms are as indicated.

A pitfall of *k*-means clustering algorithm is that it forces every spike-like signal to be affiliated with a cluster, while the total numbers of clusters are subjectively determined. This embedded a possibility that the spike waveforms in a cluster may not be homogenous. To further decipher whether a cluster truly contained waveforms of homogenous features, T^2^ distance of each data point to the centroids of each cluster was evaluated statistically. Figure 6 shows an example using PCA to select homogenous spike waveforms. The algorithm selected the data distributed near the core; these data points were collectively defined as T^2^-selected spike waveforms. On average, tentative outliers recognized as T^2^-unselected data were found in 7.0 ± 0.4% of the data points in the *k*-means clusters.

**Figure 7:**
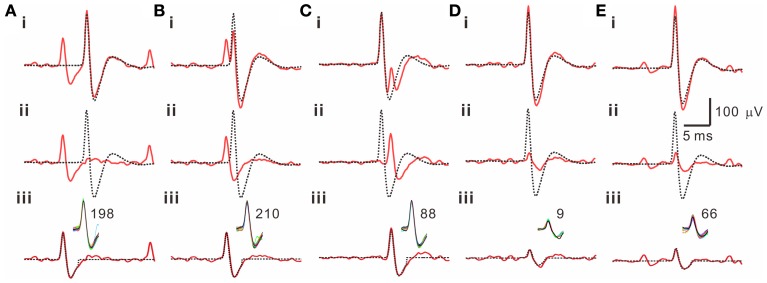
**Retrieval of T^2^-unselected waveforms by a subtraction algorithm (SA)**. 5 examples are illustrated in panels **(A–E)**. Traces in each panel are the T^2^-unselected waveforms [solid lines in **(i)**], the averaged T^2^-selected waveforms [dashed lines in **(i, ii)**], the residual waveforms acquired by a waveform subtraction in **(i)** [solid lines in **(ii, iii)**], and a retrieved waveform obtained from averaging those similar to the residual waveforms in the original recordings [dashed lines in **(iii)**]. Inset in **(iii)** shows superimposed traces of the similar waveforms retrieved by SA from a 5-min epoch of the original recordings; the numbers of superimposed traces are indicated. Panels **(A**–**C)** also demonstrate that the T^2^-unselected waveforms are decomposed into an averaged T^2^-selected waveform compounded by a spike waveform that appears prior to **(A, B)** or immediately after **(C)** the T^2^-selected waveform.

**Figure 8:**
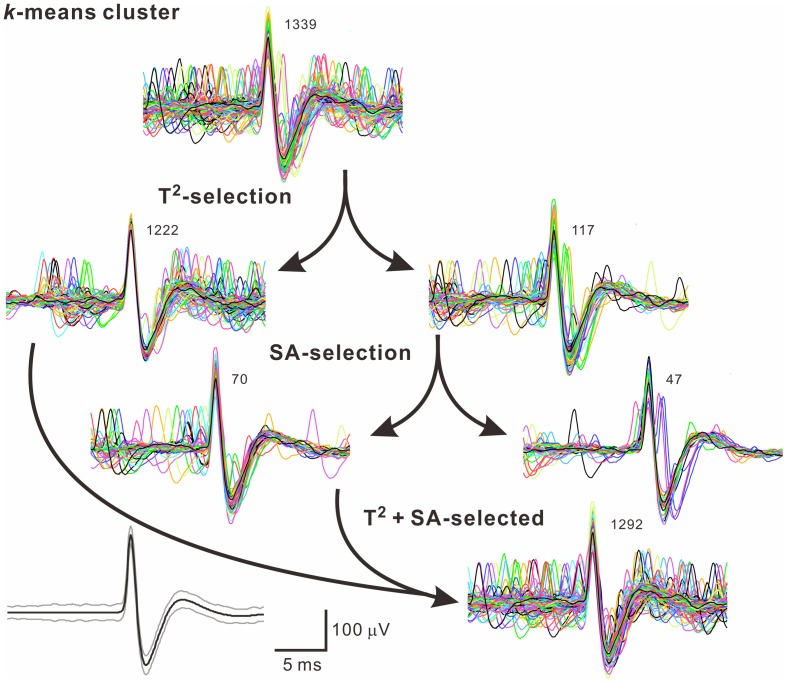
**A flow chart illustrates how to acquire unit activities**. Arrows indicate categorization of waveforms by different algorithms. Numbers of superimposed traces are as indicated. Panel in the lower left shows the averaged spike waveform (black line) and its 99% confidence limits (gray lines). In this example, 47 of 1339 (3.5%) spike waveforms in the *k*-means cluster were excluded.

Because often there are data distributed in the shell of a cluster, whether the T^2^-unselected waveforms were true outliers was further evaluated by a data-based SA. We assumed that the outlier waveforms were otherwise the ideal waveforms being distorted by the other signals that might appear independently and frequently during the recordings. A residual waveform was then acquired by subtraction of the averaged T^2^-selected “ideal” waveform from each T^2^-unselected “outlier” waveform. Figure 7 demonstrates using SA to decompose the outlier waveforms into the ideal waveforms and the residual waveforms. Appearance of these residual waveforms in the original recordings was used as a criterion to verify if they were interference signals. Original recordings contained signals of waveforms that were similar to the residual waveforms (see criteria in METHODS 2.6) were found in 99 of the 102 T^2^-unselected data groups. On average, SA retrieved 64.5 ± 2.9% of T^2^-unselected waveforms as they were considered as false outliers. In combining SA-retrieved with T^2^-selected waveforms, 97.3 ± 0.3% of the waveforms per data cluster were recognized as those sharing the same waveform features and collectively defined as unit activities, which were tentatively taken as the activities generated from a single fiber. Figure 8 is a flow chart summarizing the computational steps required for obtaining unit activities.

**Figure 9:**
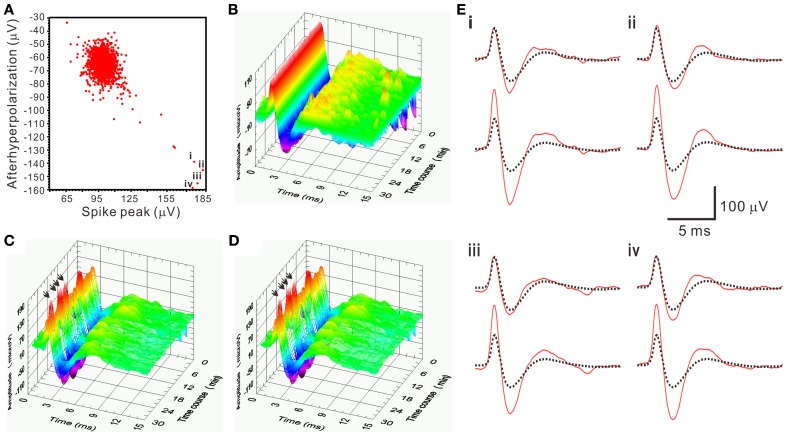
**An example demonstrating a pitfall of SA in mistaking apparently different spike waveforms. (A)** 2-D waveform feature plot of afterhyperpolarization against spike peak. The feature plot shows a concentrated data distribution in the upper left; few scattered in the lower right. Data points “**i–iv**” have the spike peak and the afterhyperpolarization with magnitudes that are nearly double the ones in those data concentrated in the upper left. Spike waveforms of the data points “**i–iv**” are shown as arrows indicated in **(C–D)** and as those solid lines in the lower panels of **(E)**. **(B**–**D)** 3-D plots of spiking events along the time course. **(B)** T^2^-selected waveforms. **(C)** T^2^-unselected waveforms. **(D)** SA-retrieved waveforms. **(E)** The residual waveforms (solid lines in the upper panels) are similar to the averaged T^2^-selected waveforms (dashed lines in both upper and lower panels), which explained a pitfall of SA in mistaking the apparently different spikes as arrows in **(C,D)** indicate.

There was a pitfall using SA. When the spike amplitude of a waveform was about twice of the averaged T^2^-selected waveform, SA would yield a residual waveform similar to the T^2^-selected waveforms. Because original recordings would contain the T^2^-selected waveforms, the similar residual waveforms could then be mistaken as a true interference signal and not be rejected as a true outlier. This led to a false-positive assignment of the unit activity by including an apparently different waveform. This error was easily detected by visualizing all the retrieved waveforms. In the analyses of all the spiking events using SA, only 5 of 1566 spiking events in one unit activity as shown in Figure 9 were considered as false-positive, which was then manually corrected.

### Accuracy of spike retrieval

**Figure 10:**
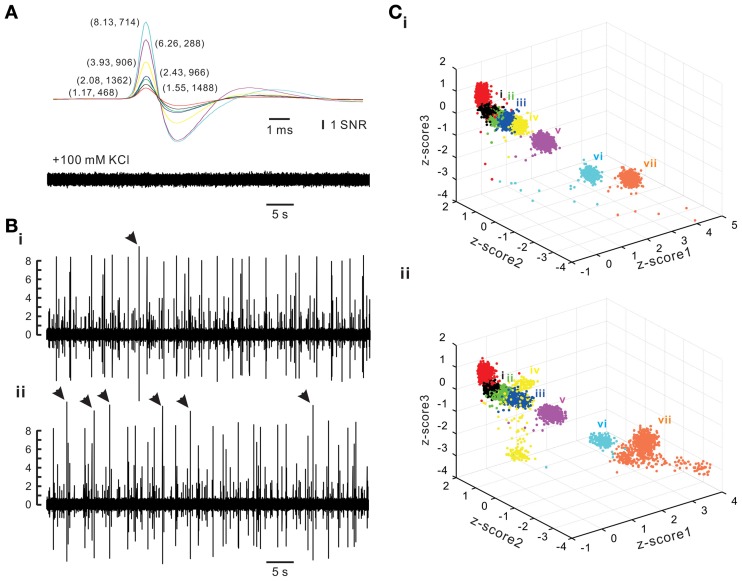
**Synthetic spiking data with or without synchronous activities between different spike groups**. Synthetic data simulating a real recording as shown in Figure 5 were generated (Appendix D). **(A)** Spike templates (upper panel) and background noise (lower panel). Averaged spike waveforms of the real data were used as spike templates. The background noise was acquired after blocking spike generation by adding 100 mM KCl into the bath solution. Spike amplitudes were expressed in units of signal-to-noise ratio (SNR). Numerical values in parentheses are the amplitudes of the spike peaks and how many spikes in that group are randomly inserted into a 30-min epoch of synthetic data. **(B)** Traces of synthetic spiking of asynchronous activities **(i)** and synchronous activities **(ii)**. Arrow heads indicate the complex spike waveforms of exceptionally large peak amplitudes resulting from overlapped spike templates. **(C)** *k*-means clustering of spiking without synchronous **(i)** or with synchronous activities (**ii**). *Z*-score1–3 are normalized values of peak amplitude, peak roundness, and afterhyperpolarization. Cluster color coded in red was discarded for further analysis because it was a collection of the afterspike potential waveforms of Clusters “vi” and “vii.” Cluster “iv” and “vii” were selected as the two with synchronous spiking activities, which yielded a dispersed distribution of data points affiliated with these clusters.

**Figure 11:**
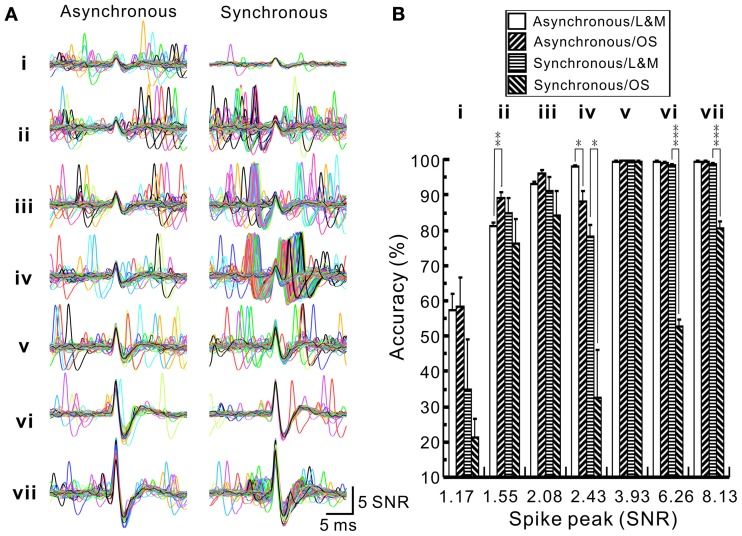
**Accuracy of spike sorting using programs written by LabVIEW and Matlab softwares (L&M) or using Offline Sorter (OS). (A)** Superimposed traces showing the waveforms retrieved by L&M protocols from the synthetic spiking data with or without synchronous activities. **(i–vii)**, spike groups. Compared to asynchronous ones, clusters iv and vii shows many overlapped waveforms resulted from their synchronous activities. **(B)** Plot of the sorting accuracy against the spike peak amplitude. Statistical analyses were based on two series of synthetic data with each containing five data sets, as one data set of each series shown in Figure 10. Accuracy of spike sorting resulted from L&M protocols is spike peak-amplitude-dependent and relatively resistant to the interferences of synchronous activities, showing that spike peaks of amplitudes greater than 3.93 SNR yield a nearly 100% sorting accuracy. In contrast, accuracy of spike sorting resulted from using OS was largely comparable to the one using L&M protocols in sorting asynchronous data but the accuracy dropped significantly in sorting synchronous data. Asterisks indicate significant differences of sorting in using L&M and OS. Student's *t*-test: ^*^P < 0.05; ^**^P < 0.01; ^***^P < 0.001.

To evaluate the accuracy of spike retrieval using our algorithms, two series of synthetic data that simulated the real recording of seven spike groups as shown in Figure 5 were generated (Appendix D). Each series contained five data sets with or without synchronous spiking activities of groups iv and vii. Figure 10 illustrates two data sets with one containing mainly asynchronous spiking and the other having substantial amounts of synchronous spiking. We also compared the accuracy of spike retrieval using our LabVIEW- and Matlab-based programs (L&M) with that using Offline Sorter (OS, v3.3.1, Plexon Inc.). For generalization, spike amplitudes were expressed in units of signal-to-noise ratio (SNR). Because the afterspike potentials of the clusters vi–vii and the spike peak of cluster i were of similar magnitudes (Figure 10A), the spike sorting required a predetermined selection of eight rather than seven centroids for *k*-means clustering (Figure 10C). Figure 11 illustrates the spike groups retrieved using our L&M protocols and the accuracy of spike retrieval compared with that using OS (see Supplementary Material I). Overall, the accuracy of spike sorting was positively related to the spike amplitudes. For spikes with peak amplitudes >3.9 SNR, nearly 100% of the spikes were retrieved using L&M, be the data containing synchronous activities or not. While the accuracy of spike retrieval using OS was largely comparable with the one using L&M in sorting synthetic data containing mainly asynchronous spiking, it dropped dramatically in that containing synchronous spiking (Figure 11B). For instance, the accuracy of spike retrieval for cluster iv of the synthetic synchronous data obtained from L&M and OS was 78.3 ± 3.1 and 32.6 ± 13.6%, respectively (*t*-test: *P* < 0.05, *n* = 5). Noticeably, an inaccurate spike retrieval for cluster vi but not for cluster v was obtained from sorting the synthetic data containing synchronous activity of clusters iv and vii using OS. This might be caused by an interference of cluster assignments due to the presence of overlapped spike waveforms (Supplementary Material I). Clustering using our L&M protocols that yielded higher accuracy of spike retrieval was apparent as demonstrated in Figure 10C.

### Evaluation of unit activity by refractory period

To verify if the spiking events in a data cluster truly originated from a single fiber, we first examined if their ISIs violated an arbitrarily-defined refractory period 3-ms. Among the 102 data clusters, 69 data clusters did not have ISIs <3-ms, 8 had <0.1% ISIs that were <3-ms, and 25 had >0.1% ISIs that were <3-ms. We further examined if the incidence of <3-ms ISIs was incurred by SA. Indeed, in 12 data clusters that all the ISIs acquired from T^2^-selected waveforms were >3-ms, addition of the SA-retrieved waveforms incurred 0.24 ± 0.07% of ISIs that were <3-ms, indicating a potential risk of retrieving false-positive waveforms in using SA. The minute amounts of false-positive spiking events were easily corrected manually by removing the outliers that had larger T^2^ distances. For simplicity, unless otherwise mentioned, only the data clusters originally containing <0.1% ISIs that were <3-ms (*n* = 77) were included in the following evaluation.

### Evaluation of unit activity by multimodal gaussian analysis of ISI probability distribution

Spontaneous spiking of SPNs under our experimental conditions reveals an ISI probability distribution that is well-described by Gaussian functions (Su et al., 2007). Because most splanchnic nerve fibers are the projecting axons of SPNs, we sought to determine if the unit activity recorded in this study had the same features. Moreover, to avoid the shadowing effects that lead to an abrupt absence of ISIs at <3-ms as a result of failure in detecting overlapped spikes (Bar-Gad et al., 2001), i.e., a false identification of the refractory period, whether the ISI probability curves “declined” promptly toward an ISI range in the refractory periods was also used as a criterion to evaluate if the unit activity truly originated from a single fiber.

**Figure 12:**
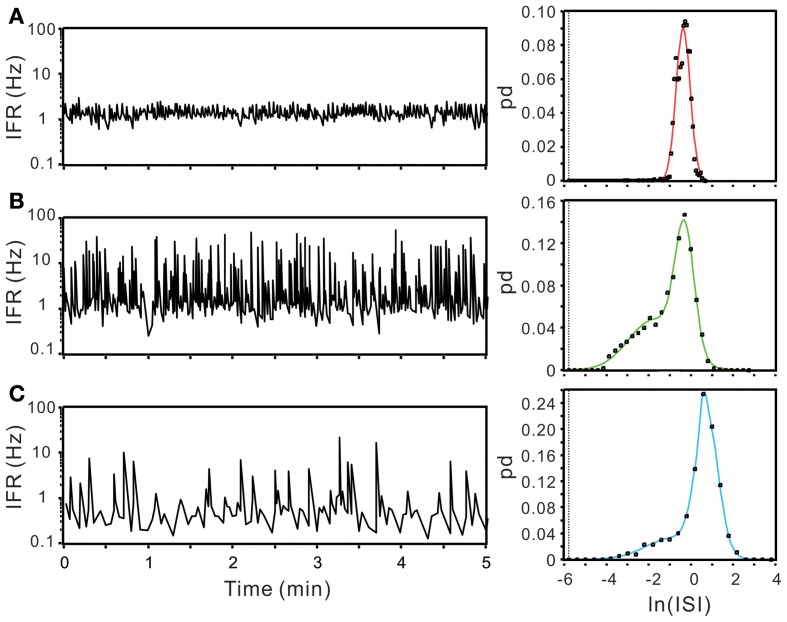
**Using ISI probability curves to evaluate unit activities**. Panels **(A–C)**, examples from three different experiments. In each example, plots show the fluctuation of instantaneous firing rate (IFR, the left panels) and the ISI probability distribution (dots, the right panels). Solid lines in the ISI probability plot depict the curve fitting by Gaussian functions of unimode **(A)**, bimodes **(B)**, and trimodes **(C)**. All the three examples demonstrate a leftward decline of ISI probability distribution. pd, probability density. In this and following figures, the dashed vertical lines in ISI probability plots crossing the *x*-axis at e^−5.809^ s indicate the 3-ms refractory period.

**Figure 13:**
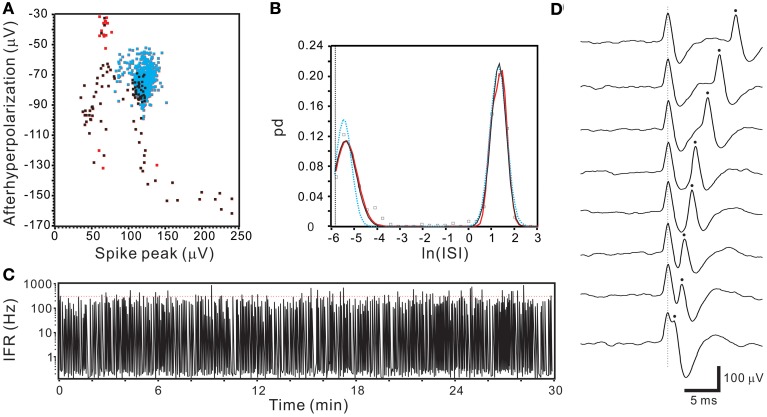
**Segregated ISI probability distribution obtained from a unit activity with overlapped spikes**. **(A)** 2-D waveform feature plot showing a continuum of data distribution. Dots in blue, black, and red represent the data points of T^2^-selection, SA-selection, and excluded outliers. Note the data distributed at the right lower extreme have parametric values (~230, −150) nearly double those concentrated in the center (the centroid of blue dots: ~120, −70). **(B)** Plots of ISI probability distribution. Analyses include data of T^2^-selection only (blue), T^2^-and SA-selection (black), and T^2^-/SA-selection plus outliers (red). All curves show double peaks. One peak was centered around 4.66 ms (i.e., e^−5.369^ s) with a probability distribution extending into the ISI range that violated the refractory period. **(C)** IFR time series plot of T^2^- and SA-selected data showing aberrant firing of exceedingly high IFR. Red dashed line indicates IFR = 300 Hz. **(D)** Original recording traces showing overlapping of two spikes with similar waveforms, explaining the continuous variation of parametric values in **(A)** as a result of spike overlapping at different phases.

ISI probability curves were fitted by Gaussian functions with different number of modes (Figure 12). To evaluate how many modes provided the best fitting, AIC_c_ obtained from each curve fitting were compared. Table 1 summarizes the criterions of selecting Gaussian models for ISI probability curve fitting. Because AIC_c_ describes the entropy of error estimates from a model, the model that generates a minimal AIC_c_ was considered the best. Substantial difference of AICc (ΔAIC_c_) was obtained from Gaussian fitting using different numbers of modes, and thus, an appropriate selection of the particular Gaussian modes was achieved accordingly (Table 1). Among the 77 ISI probability curves, 10 (13%) were best fitted by a unimodal Gaussian distribution, 22 (29%) fitted by bimodal Gaussian, and 45 (58%) fitted by trimodal Gaussian. All together, 189 Gaussians were obtained from the fitting of 77 ISI probability curves; the median of all the Gaussian modes was 1.11 s, which is comparable to that previously observed in the data obtained from patch-clamp recordings of SPN spiking activities (Su et al., 2007). Moreover, all the ISI probability curves consistently showed a leftward exponential decline of probability in the lower ISI range, reflecting a diminution of firing probability in an exponential decay manner during the relative refractory periods (Figure 12). By contrast, Figure 13 shows an example of ISI probability curve with two distinct modes at 5.3 ms and 4.2 s; this unit had 2.2% ISIs that were <3-ms. This unit activity was considered as a combined activity of two fibers with very similar spike waveforms. In summary, the unit activities that displayed Gaussian firing properties and had ISIs not violating refractory periods were considered as activities originated from single fibers.

**Table 1:** **Statistical features of ISIs and evaluation of multimodal Gaussian curve fitting of ISI probability distribution by corrected Akaike information criterion (AIC_c_)**.

**Statistical features**	**Gaussian models**		
	**Unimodal**	**Bimodal**	**Trimodal**
Mean ISI (s)	5.719 ± 1.748	1.359 ± 0.207	2.692 ± 0.524
Minimal ISI (s)	0.070 ± 0.041	0.066 ± 0.032	0.146 ± 0.045
*Adjusted r*	0.9823 ± 0.0054	0.9683 ± 0.0087	0.9695 ± 0.0040
ΔAIC_c2-1_	11.6 ± 1.8	−42.3 ± 6.4	−17.2 ± 4.6
ΔAIC_c3-1_	26.6 ± 5.4	−26.6 ± 7.2	−54.0 ± 7.5
ΔAIC_c3-2_	15.0 ± 4.7	15.7 ± 3.0	−36.8 ± 9.4
*n*	10	22	45

Analyses were based on data collected from 77 unit activities. ISI probability curves were fitted by Gaussian equation: $y=\sum_{i=1}^{k}a_{i}\cdot e^{\left\{-0.1/2{\left[(x-b_{i})/c_{i}\right]}^{2}\right\}},$ where *k* is 1, 2, or 3 representing for uni-, bi-, or trimodal Gaussian, respectively. The Gaussian that generated a minimal AIC was selected as the best model for ISI probability curve fitting. ΔAIC_c_ are the subtraction values of the AIC obtained from Gaussian fitting using different number of modes; the subtractions between Gaussian modes are as the subscript numbers indicated.

### Evaluation of unit activity by determining the change of waveform features as a function of their preceding ISIs

**Figure 14:**
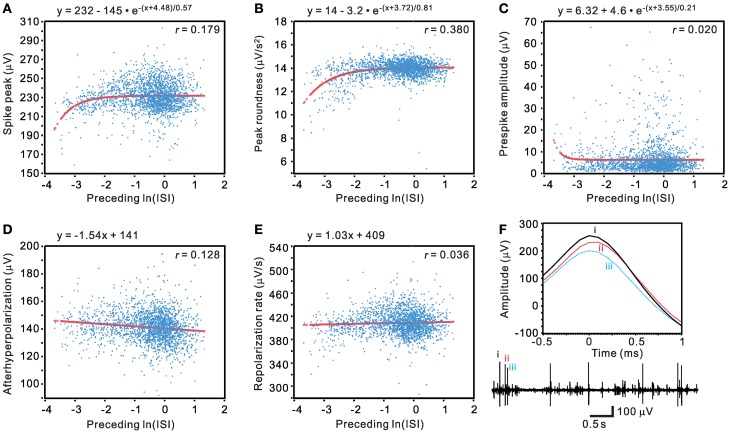
**Evaluation of unit activity by determining if a change of spike waveform features is a function of their preceding ISIs. (A–E)** Plots of waveform features against their preceding ISIs in a normal-log scale. Curve fitting of the data distribution used exponential relaxation functions **(A–C)** or linear functions **(D–E)**, selected by ΔAIC_c_. Thick lines are the simulated curves. *r*, adjusted regression coefficient. Tests of goodness of fit: *P* < 0.001 in **(A–B)** and **(D)**; *P* <0.05 in **(C)** and **(E)**. **(F)** Original traces showing preceding ISI-dependent change of spike waveforms. Lower panel, the original trace. Upper panel, the spikes “i–iii” as shown in the original trace are aligned at their peaks (0-ms) to demonstrate an attenuated peak amplitude in spikes with short preceding ISIs.

Traditional waveform-based spike sorting that does not consider spiking history could have flaws (Pouzat et al., 2004; Ventura, 2009). This is largely because spike amplitudes and shapes are not stationary and may be influenced by their preceding spiking events. We sought to determine if a waveform parametric value was a function of its preceding ISI. Five waveform features, including spike peak, peak roundness, prespike amplitude, afterhyperpolarization, and repolarization rate, were plotted against their preceding ISIs in a normal-natural log scale to manifest a change of waveform features in the range of short ISIs (Figure 14). The pattern of data distribution and the extent of correlation with their preceding ISIs varied between different waveform parametric plots. By visual inspection, waveform parametric values were positively, negatively, or not correlated with their preceding ISIs. Figure 14 shows an example that the waveform features are described as an exponential or a linear function of their preceding ISIs. The choice of exponential vs. linear equation for curve fitting was largely based on which equation could yield a lower AIC_c_, with the exceptions when goodness of fit was not achieved by fitting using the equation that attained a lower AIC_c_. Tests of goodness of fit among the 77 units activity showed that 71 units had at least one of the five waveform features failed in curve fitting by the exponential or the linear equation, seven units had all the five waveform parameters fitted by the equations, and six units had all the five parameters failed in curve fitting. Among the five waveform features, analyses of spike peak amplitudes demonstrated that only 28 out of 77 units, the least number of units, failed in curve fitting. Thus, for simplicity, the spike peak amplitude was chosen as one of the most sensitive waveform features to evaluate if its change was preceding ISI-dependent.

**Figure 15:**
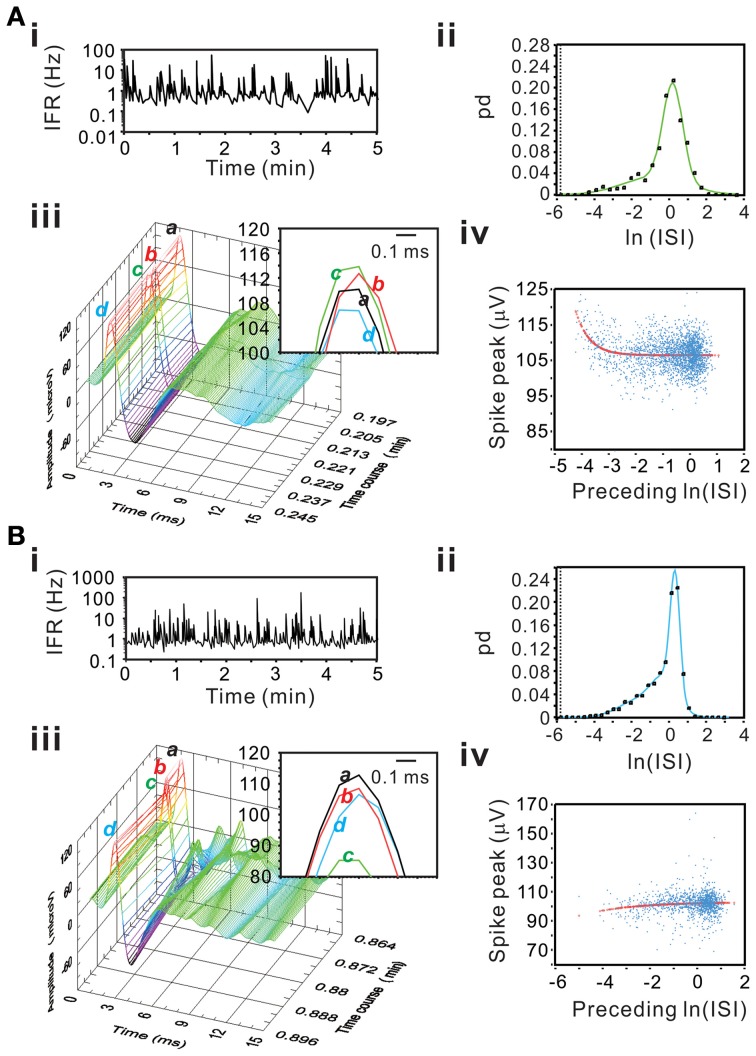
**Examples showing an incremental (A) or a decremental (B) change of spike peak amplitudes as a function of their preceding ISIs**. Panels **(A,B)** are examples from different experiments. **(i)** IFR time series plot. **(ii)** ISI probability plot; the square symbols are the original probability distribution and the solid line are the curves obtained from Gaussian fitting using bimodes **(A)** or trimodes **(B)**. **(iii)** 3-D plot of spike occurrence; waveform traces in inset “*a*–*d*” shows spike peaks. In both panels **(A)** and **(B)**, spike “*c*” has the shortest preceding ISI; it has the highest peak amplitude in **(A)** and the lowest peak amplitude in **(B)**. **(iv)** Plot of spike peak amplitude against preceding ISI. Peak amplitudes increase **(A)** or decrease **(B)** as a function of preceding ISI in an exponential relaxation manner.

Figure 15 show examples of incremental or decremental changes of spike peak amplitudes as a function of their preceding ISIs. Among the 77 units, short preceding ISIs having augmented peak amplitude were observed in 18 units fitted by the exponential equation and the other 18 units fitted by the linear equation, whereas short preceding ISIs having attenuated peak amplitude were observed in 8 units fitted by the exponential equation and the other five units fitted by the linear equation. Therefore, a total of 49 of 77 unit activities had spike peak amplitudes as a function of their preceding ISIs. For those units best fitted by the linear equation, the slopes of their curves were generally low, indicating a weak dependence of the peak amplitudes on their preceding ISIs. In summary, using a preceding ISI-dependent change in spike peak amplitudes as a criterion, we confirmed 64% of unit activities as the activities originated from single fibers.

## Discussion

Oligofiber activities were successfully recorded from the collagenase-dissociated splanchnic sympathetic nerve fascicles in the rat thoracic spinal cord preparations *in vitro*. Using a data-based, computational process—SA to dissect spike overlapping, we effectively reduced false outliers in the process of waveform recognition. Thus, 97% of spiking signals in a *k*-mean cluster having homogeneous waveforms were taken as unit activities. Most unit activities were considered to be originated from single fibers because of the stochastic homogeneity of waveforms, the Gaussian firing properties characterized by a declined spiking in refractory periods, and the preceding ISI-dependent changes in waveform features. By simultaneously recording several single-fiber activities, the methodology established here can tackle issues of spiking synchronicity. With some modifications, we believe that the oligofiber recording techniques is applicable to any peripheral nerve preparations aiming for detailing their single-fiber activities.

### Methodological considerations for spike sorting

This study did not provide a sophisticated mathematical solution for spike sorting. Instead, we made acquisition of single-fiber activities more feasible from recordings of collagenase-dissociated nerve fascicles. The success of this biological approach was apparent as at least one unit activity was recorded in every experiment. This achievement partially relies on a meticulous trial for the experimenters using small caliber micropipettes to sample fibers generating spikes of distinguishable magnitudes, rather than on an ingenious mathematical process of spike waveforms. Nonetheless, being aided by series of LabVIEW- and MATLAB-based computer programs for spike sorting, we could extract spikes of distinct waveform features and confirm their spiking activities as those originated from single fibers.

It may be argued that developing the custom-made programs is a futile effort when many commercial softwares for spike sorting are available. This is not fundamentally true as an apparent advantage using our own programs is the transparency of the sorting process as suggested in the report of (Lewicki, 1998). Besides, the installation cost is much lower and the utilization of these programs provides much greater flexibility to suit our experimental needs. Another advantage using these custom-made programs is the ease to quantify the amounts of potential outliers, which are further evaluated using algorithms to determine if they are the waveforms being contaminated by other interference signals (Figure 7). We notice that OS installs this function by a subjective determination of the “outlier threshold,” which does not allow users to yield an objective evaluation of the outlier waveform or to find clues for exploring how such complex waveforms occur. To our best knowledge, our process that can effectively retrieve ~65% of those dissimilar waveforms being falsely recognized as outliers is not found in any commercial available programs.

Various algorithms have been developed in other laboratories to decompose overlapped spikes for different applications (Atiya, 1992; Takahashi et al., 2003; Zhang et al., 2004; Vargas-Irwin and Donoghue, 2007; Franke et al., 2010). Most of them are based on a preprocess construction of waveform templates to determine if a complex waveform results from overlapping of these templates. In fact, this turns to be a limitation of these algorithms in that they can only decompose complex waveforms based on predetermined templates. Many interference signals may not be readily detectable because of low magnitudes or low incidence (e.g., Figure 7D). It is noticeable that there are very inactive neural activities that barely form data clusters (e.g., Cluster “ii” in Figure 3). These neural activities being inactive yet not completely quiescent are unlikely to be recapitulated as waveform templates. Moreover, nonstationarity of background noise or subthreshold spiking signals can be the other sources of confounding signals adding to the otherwise ideal waveforms. All these undetected biological or non-biological interferences complicate an application of the conventional algorithms in tearing the overlapped spikes apart. Our algorithms for complex waveform decomposition directly taking the data obtained from original recordings as reference have no such limitation.

How accurate is our methodology for spike sorting? An easy answer for this question is usually not available for any spike sorting algorithms in dealing with the real data. To surmount this inherent difficulty, we used a synthetic data that simulated the real recordings. We found that the accuracy of spike sorting using our protocols was largely related to spike amplitudes, be it with or without synchronous activities. While it is clear that acquisition of spiking signals with high SNR values is fundamental to spike sorting, our simulation approaches also imply that, for signals obtained from single-electrode recordings, an overemphasis on computational algorithms may be futile and risky when spike sorting was aiming to signals of amplitudes <2 SNR. Noticeably, in our simulation tests, stationarity of the spike waveforms and independent assortment of spiking activities between different spike groups were assumed. In real recordings, a substantial change in the spike waveforms (e.g., Figures 14, 15), the extent of waveform similarity between different fibers (e.g., Figure 13) and the synchrony in spiking may further complicate the spike sorting and diminish its accuracy.

As a focus on biological approaches, the feature extraction of spike waveforms in our hand did not totally depend on a blind PCA that made biophysical interpretation of PCs impossible. The waveform features were empirically determined by the parametric values obtained from discrete segments of the waveforms that might implicate distinct biophysical meanings. For instance, because the proximity of fibers to the recording electrode is fairly constant using the suction electrodes in this study, the spike maximum could reflect the nodal action currents, which might vary between fibers of different sizes (Marks and Loeb, 1976; Kovac et al., 1982). The rate of potential change during the rising or decaying phase of the waveforms could reflect the electronic charging or discharging time constant that also differed between fibers of various diameters. Indeed, similar to the process as we took here, it has been shown that taking the first derivatives of the partial spike waveforms as a feature can improve spike feature extraction (Yang et al., 2009). Moreover, the prespike amplitude and the magnitude of afterhyperpolarization could reflect the intrinsic properties of fibers, and thus, being considered as another distinguishable feature. In summary, spike sorting algorithms used here were based on some distinct parameters that could best describe the biophysical features of individual fiber, rather than a pure waveform-based analysis. Besides, using the waveform features for spike sorting could minimize the computational costs and make data clustering more efficient (Figure 5).

### Grouping unit activity by combining T^2^-selected homogeneous spike waveforms with SA-retrieved nonhomogeneous waveforms

On the variability of manual spike sorting, average error rates of 23% false positive and 30% false negative has been noticed (Wood et al., 2004). To minimize errors, we established a semi-automatic algorithm. Our classification of spikes was largely based on the stochastic features of waveforms using T^2^ distance to extract homogenous waveforms in a *k*-means cluster followed by retrieving nonhomogeneous waveforms using SA. It should be emphasized that SA did not provide a singular solution. As an analogy to linear algebra, signals obtained from a single micropipette are the outcomes of an underdetermined recording system that is compounded by more than one unknown signal sources. The solution for a system as such cannot be independent. In other words, there could be multiple solutions for an underdetermined system. We surmount this mathematical obstacle by taking a biological approach and allowing a computer program to match the spike residuals that might occur during the recording period. If the events indeed happened, the spike residuals were considered to be a true interference to the otherwise homogeneous waveforms. This algorithm was effective in retrieving dissimilar waveforms and could minimize false negative data. In this study, this approach actually retrieved ~65% of the T^2^-unselective waveforms and helped to confirm ~97% of the spiking events in a *k*-mean cluster as unit activities.

### Evaluation of unit activities as originated from single fibers

Spike waveforms are not stationary and are likely to be affected by their preceding spiking events. For example, spike amplitude tends to decrease at high discharge rate (Fee et al., 1996; Zhang et al., 2007), probably because of limited availability of Na^+^ channels during fast spiking (Miles et al., 2005). We evaluated if a unit activity truly originated from one single fiber primarily by two methods. One was seeking the evidence for an influence of refractory period in the ISI probability curve, the other was determining whether the parametric values of waveform features changed as a function of their preceding ISIs.

On the ISI probability curves that decline toward the refractory period, we found that all the 77 unit activities could originate from single fibers. On determining a preceding ISI-dependent change of waveform features, we confirmed that 71 of 77 unit activities originated from single fibers because at least one of their spike waveform features changed as a linear or exponential function of their preceding ISIs. Why some units did not display ISI-dependent change in some of their waveform features were not systemically investigated. It seems that, most of them have relatively small magnitudes of spike potentials with SNR <2 or appear to be less active in having lower numbers of spike potentials (<200 spike potentials in 30-min epoch). Some were characterized by a highly centered ISI probability distribution with spiking rarely falling into the short ISI ranges. No violation of the refractory periods was observed in the units lacking an ISI-dependent change in their waveform features. Therefore, the preceding ISI-dependent change of waveform features could help to verify the unit activities as originated from single fiber, but was not sufficient as the sole criteria to confirm a single-fiber activity.

The recording noise can be used as a signature to evaluate the quality of spike sorting and help the experimenters to judge if the variation of waveforms could be simply attributed to the white noise during recording (Pouzat et al., 2002). This type of analysis is based on the assumption that spike waveforms are stationary. In this study, we have demonstrated that many of the spike waveform features change as a function of their preceding ISIs. As estimated by the standard deviation of recorded signals and compared to the prespike signals, the nonstationarity of spike waveforms renders a greater signal variation during the spike occurrence (data not shown). The nonstationarity of spike waveforms makes the use of recording noise as a mean to evaluate spike sorting difficult.

### Not all axonal spike peak amplitudes attenuate during fast firing

Although spike peak amplitude is a sensitive waveform parameter to reveal a preceding ISI-dependent change, not all units have their spike peak amplitudes attenuated during fast firing. Among 49 units with peak amplitudes correlated with ISIs, augmented and attenuated peak amplitudes during fast firing were found in 36 units and 13 units, respectively. This observation was not expected. We did not have clear clues regarding why some axonal spike peak amplitudes augment following shorter preceding ISIs. Nonetheless, it is possible that axonal firing properties are different from those of somatodendrites (Shu et al., 2007; Kress and Mennerick, 2009; Sasaki et al., 2011). Experiments in rat hippocampal neurons or neocortical pyramidal neurons show that axonal spikes are more resistant to amplitude reduction than somatic spikes during brief spike trains (Williams and Stuart, 1999; Meeks et al., 2005). Compared to dendrites, axonal conduction has higher safety factors that guarantee a high-fidelity of action potential propagation (Mackenzie and Murphy, 1998). While the involvement of various channels in axonal spike propagation to account for ISI-dependent waveform variability remains to be explored, the inconsistency in changes of waveform features as a function of their preceding ISIs diminishes the applicability of taking preceding ISI as another variable for axonal spike sorting.

## Conclusions

The feasibility of oligofiber recording techniques and computational processes enables us to examine the spiking behaviors of several sympathetic fibers, simultaneously. Using a data-based SA process that intuitively resolves overlapped spikes, we reclaim spikes of seemingly dissimilar waveforms. Our computational algorithms by minimizing false negative data may help to restore the fidelity of rate coding embedded in contiguous spiking events. This is especially crucial for information coding in SND, wherein effective commands are likely encoded in patterns of synchronous bursts.

### Conflict of interest statement

The authors declare that the research was conducted in the absence of any commercial or financial relationships that could be construed as a potential conflict of interest.

## Appendix A: *k*-means clustering

*Z*-scores (*z*) of the six waveform feature parameters obtained from *n* spikes occurred in an epoch of continuous recording were calculated by

$$z_{ij}=(w_{ij}-{\bar{w}}_{j})/s_{j}$$

where *i* = 1 to *n* index of spike data, *j* = 1 to 6 index of the waveform parameter (*w*), and *s*_*j*_ the *j*th standard deviation of *w*_*ij*_, calculated by

$$s_{j}={\left[\frac{1}{n-1}\sum_{i=1}^{n}\left(w_{ij}-{\bar{w}}_{j}\right)\right]}^{1/2}$$

This calculation normalized the weight of each waveform parameter and gave a set of *n* data points in ℜ^6^. For comparisons, in some experiments, *z*-values in a space of ℜ^32^ were also acquired by taking account of the spike waveform segments between 1.2-ms prior to and 2-ms after the spike peak, i.e., the parametric values of the spike waveforms without feature extraction. An integer *k* was empirically selected; *k*-means clustering determined a set of *k* centroids in ℜ^6^ or ℜ^32^. Each centroid is the component-wise median of the points in that cluster, so as to minimize the sum of distance from each data point to its nearest centroids:

$$\sum_{i=1}^{n}d(i,c(i))$$

where *d*(*i, c*(*i*)) is the cityblock distance that measures the dissimilarity of a data point *i* to its nearest centroid, denoted by *c*(*i*), and is defined as:

$$d(i,c(i))=\sum_{j=1}^{D}\left|i_{j}-c_{j}(i)\right|$$

where *D* is the dimension of ℜ^6^ or ℜ^32^, *i*_*j*_ and *c*_*j*_(*i*) are the *j*th-components of the two points. For comparisons, instead of using cityblock distance, squared Euclidean distance was used for *k*-means clustering in some experiments. Therein, each centroid is the mean of the data points in the cluster. The computation of *k*-means clustering utilizes an iterative algorithm to minimize the sum of distances. Computation began from randomly-selected initial centroids, followed by assigning every point to its nearest cluster and calculating the medians of each cluster. The acquired medians were then used as the new values of centroids. The computation repeated until the sum of distance converged.

## Appendix B: silhouette values

The silhouette value for each point *i* is a measure of the similarity of a point to points in its own cluster compared to points in other clusters. Computation was based on the following definition:

*a*(*i*) = the average distance of point *i* to the other points in its cluster *A*.

*d*(*i, C*) = the average distance of point *i* to points in other clusters *C*.

$b(i)=\underset{C\neq \;A}{\min}d(i,C)$

The silhouette value is defined as:

$$s(i)=\frac{b(i)-a(i)}{\max\{a(i),\;b(i)\}}$$

Therefore, the silhouette values range from −1 to +1 for each point *i*. Those points with silhouette +1 indicates that they are far from the neighboring clusters; 0 indicates that they are not distinctly in one cluster or another; −1 indicates that they may be assigned to the wrong cluster.

## Appendix C: principal component analysis

Principal component analysis (PCA) is effective for simplifying data representation in multivariate space. First, *z*-scores of the six waveform parameters for the *n* spikes appeared in 30-min epoch were calculated and defined as a 6 × *n* data matrix **X** with zero empirical mean. In the matrix, each row corresponds to a particular type of measurements; each column corresponds to the spike detected at a moment during the recording. Second, a unit vector along whose direction the variance in **X** is maximized in ℜ^6^ was found and this vector was saved as basis **p1**, i.e., the first principal component (PC). Third, another unit vector that is orthogonal to all previous selected bases and along whose direction the variance is maximized was found and the vector was saved as basis **pi** (i.e., the *i*th PC). Fourth, the third procedure was repeated until six bases were found. Finally, an ordered set of **pi**, i.e., the principal components of **X**, and the eigenvectors of $C_{X}\equiv \frac{1}{n}XX^{T}$ were obtained. By putting these 6 principal components in the rows of orthonormal matrix **P**, we could get **Y** = **PX** such that $C_{Y}\equiv \frac{1}{n}YY^{T}$ is a diagonal matrix. Therefore, by a linear combination, the *z*-scores that represented the original features of spike waveforms were transformed into 6-D nonparametric PCs. The Hotelling's T^2^ distance for each spike can be obtained as *T*^2^ = *n*(*P* − *m*)'*S*^−1^(*P* − *m*), where n is the number of spikes, *P* is the matrix of PCs, m is the mean vector and *S*^−1^ is the inverse of the covariance matrix.

## Appendix D: synthetic data

Synthetic spiking data simulating a real recording as shown in Figure 5 was generated by custom-made programs. First, spike templates in a waveform segment 12-ms prior to and 13-ms after the spike peaks were acquired by averaging the homogeneous waveforms of those from T^2^-selected data. Second, the background noise signals were obtained from the original recording after 100 mM KCl was added into the bath solution for depolarization-blockade of spike generation. Synthetic spiking mimicked the mean firing behaviors of different fibers. The firing period of a spike group or a fiber was determined as an inverse of their average firing rate. In each firing period, except for the initial 3-ms, one spike template was added to the background noise at a randomly-selected time point. This protocol repeated until a 30-min epoch of synthetic data containing seven spike groups was produced. For each spike group, the program generated a synthetic spiking behavior that was largely tonic or asynchronous without violating 3-ms refractory periods. While being added together, some complex waveforms might occur when two or more spike templates from different groups were collided at a time point close to each other (e.g., an outlier waveform as arrow head indicated in Figure 10Bi). On the other hand, synchronous spiking data was created by shifting the spiking times of one spike group toward the spiking times of the other to generate a synchronous firing in between. We chose group iv and vii, because both had relatively high firing rates and apparently different spike peak amplitudes. First, the abovementioned algorithms that create asynchronous spiking times were used. Second, some of the spiking times in group iv, which appeared near the spiking times of group vii, were randomly relocated into a period of ≤5 ms around the spiking times of group vii. A synchronous spiking data was therefore created, which contained many complex waveforms as shown in Figure 11. Subsequently, the accuracy of spike sorting for each spike group was simply obtained from the calculation:

$$\text{Accuracy}=\left(1-\left|1-\frac{N_{\text{retrieved}}}{N_{\text{synthetic}}}\right|\right)•100\%$$

where *N* is the number of retrieved or synthetic spikes.
