# Supplementary material for: Computational solution of spike overlapping using data-based subtraction algorithms to resolve synchronous sympathetic nerve discharge
Source: Front Comput Neurosci. 2013 Oct 31;7:149. doi: 10.3389/fncom.2013.00149 (PMC3813947; doi:10.3389/fncom.2013.00149)
Supplement: Supplementary file 1 [file Presentation1.PDF]

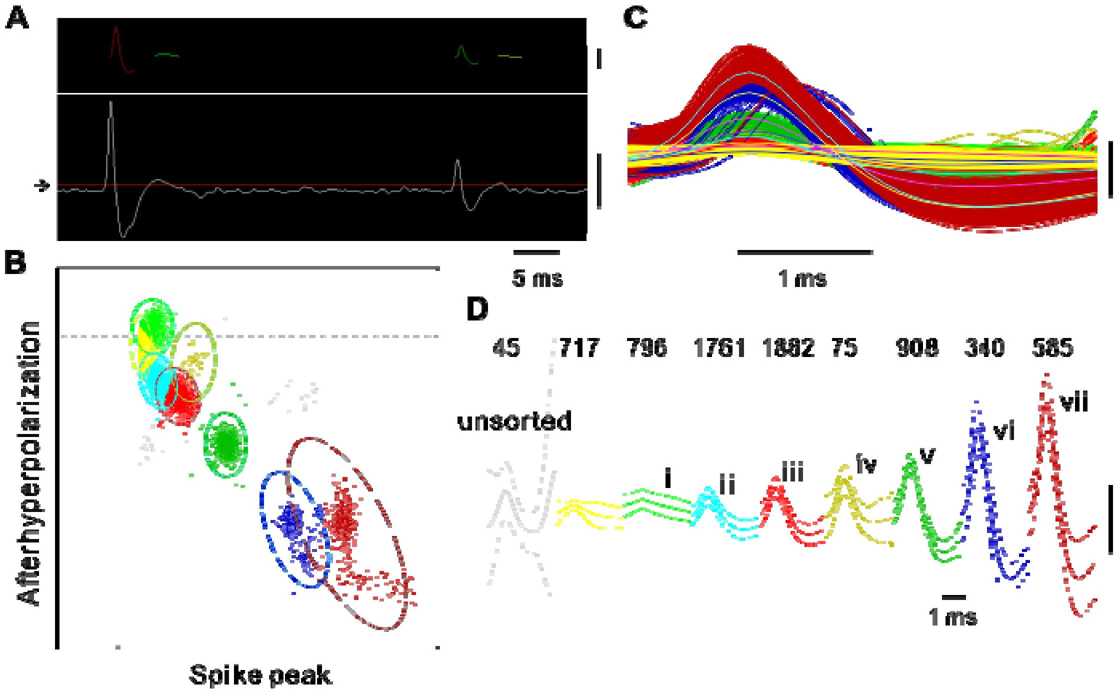

Supplementary Material I | Spike sorting using Offline Sorter (OS). Sorting was performed using the data set containing synchronous activities as shown in **Figures 10–11**. (**A**) Detection of spike potential waveforms. The arrow and the red line in the lower panel indicate the threshold for spike detection. Traces in the upper panel show detected waveforms. Some spike afterpotentials were detected and recognized as individual units. Each waveform is 3.2 ms in length with a prethreshold period of 0.8 ms followed by a dead time of 2.4 ms. Vertical bars represent 5 SNR. (**B**) Unit clusters. Units were automatically grouped into 8 clusters by *k*-means scan sorting. Dashed ellipses show an arbitrary selection of outlier threshold of 3.3, a criterion to assign unsorted to their closest units. Also in comparison with **Figure 10Cii**, misassignments of units were apparent in groups vi (blue dots, without synchronous activities) and group vii (red dots, with synchronous activities). (**C**) Superimposed spike waveforms. Units are color coded. (**D**) Unit's mean waveforms with confidence intervals. Numerical values on top indicate the numbers of spikes in a unit cluster. Compared to the spike numbers as shown in **Figure 10A**, only 75 of 966 spikes in group iv, which synchronized with group vii, were retrieved by OS.
